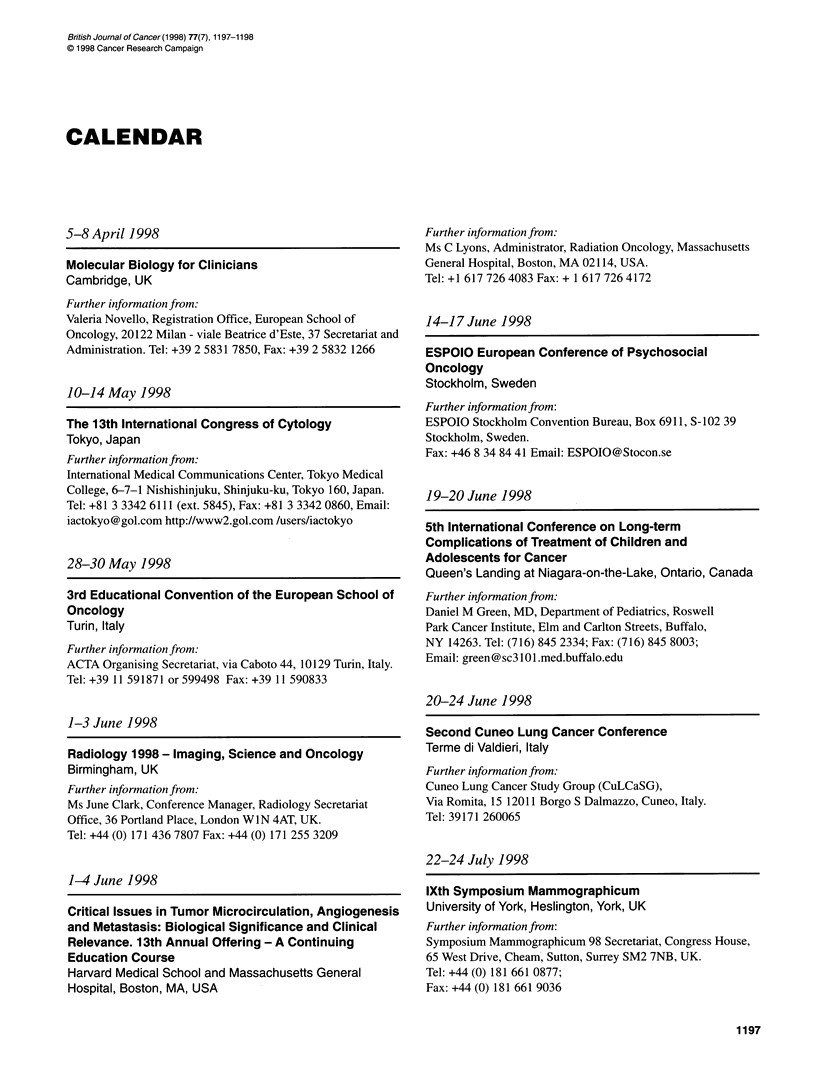# Calendar

**Published:** 1998-04

**Authors:** 


British Journal of Cancer (1998) 77(7), 1197–1198
© 1998 Cancer Research Campaign

### CALENDAR

*5–8 April 1998*

**Molecular Biology for Clinicians**
Cambridge, UK

*Further information from:*
Valeria Novello, Registration Office, European School of Oncology, 20122 Milan - viale Beatrice d'Este, 37 Secretariat and Administration. Tel: +39 2 5831 7850, Fax: +39 2 5832 1266

*10–14 May 1998*

**The 13th International Congress of Cytology**
Tokyo, Japan

*Further information from:*
International Medical Communications Center, Tokyo Medical College, 6–7–1 Nishishinjuku, Shinjuku-ku, Tokyo 160, Japan. Tel: +81 3 3342 6111 (ext. 5845), Fax: +81 3 3342 0860, Email: iactokyo@gol.com http://www2.gol.com /users/iactokyo

*28–30 May 1998*

**3rd Educational Convention of the European School of Oncology**
Turin, Italy

*Further information from:*
ACTA Organising Secretariat, via Caboto 44, 10129 Turin, Italy. Tel: +39 11 591871 or 599498 Fax: +39 11 590833

*1–3 June 1998*

**Radiology 1998 – Imaging, Science and Oncology**
Birmingham, UK

*Further information from:*
Ms June Clark, Conference Manager, Radiology Secretariat Office, 36 Portland Place, London W1N 4AT, UK. Tel: +44 (0) 171 436 7807 Fax: +44 (0) 171 255 3209

*1–4 June 1998*

**Critical Issues in Tumor Microcirculation, Angiogenesis and Metastasis: Biological Significance and Clinical Relevance. 13th Annual Offering – A Continuing Education Course**
Harvard Medical School and Massachusetts General Hospital, Boston, MA, USA

*Further information from:*
Ms C Lyons, Administrator, Radiation Oncology, Massachusetts General Hospital, Boston, MA 02114, USA. Tel: +1 617 726 4083 Fax: + 1 617 726 4172

*14–17 June 1998*

**ESPOIO European Conference of Psychosocial Oncology**
Stockholm, Sweden

*Further information from:*
ESPOIO Stockholm Convention Bureau, Box 6911, S-102 39 Stockholm, Sweden. Fax: +46 8 34 84 41 Email: ESPOIO@Stocon.se

*19–20 June 1998*

**5th International Conference on Long-term Complications of Treatment of Children and Adolescents for Cancer**
Queen's Landing at Niagara-on-the-Lake, Ontario, Canada

*Further information from:*
Daniel M Green, MD, Department of Pediatrics, Roswell Park Cancer Institute, Elm and Carlton Streets, Buffalo, NY 14263. Tel: (716) 845 2334; Fax: (716) 845 8003; Email: green@sc3101.med.buffalo.edu

*20–24 June 1998*

**Second Cuneo Lung Cancer Conference**
Terme di Valdieri, Italy

*Further information from:*
Cuneo Lung Cancer Study Group (CuLCaSG), Via Romita, 15 12011 Borgo S Dalmazzo, Cuneo, Italy. Tel: 39171 260065

*22–24 July 1998*

**IXth Symposium Mammographicum**
University of York, Heslington, York, UK

*Further information from:*
Symposium Mammographicum 98 Secretariat, Congress House, 65 West Drive, Cheam, Sutton, Surrey SM2 7NB, UK. Tel: +44 (0) 181 661 0877; Fax: +44 (0) 181 661 9036